# The Discovery of an Intracardiac Thrombus Following a Mild COVID-19 Infection: A Case Report and Review of Literature

**DOI:** 10.7759/cureus.39745

**Published:** 2023-05-30

**Authors:** Jenish Bhandari, Anas M Abbas, John J Alfarone, Syed Huda, Niranjan Ojha

**Affiliations:** 1 Medicine, Upstate University Hospital, Syracuse, USA; 2 Medicine, Norton College of Medicine, Upstate University Hospital, Syracuse, USA; 3 Cardiology, Upstate University Hospital, Syracuse, USA

**Keywords:** cardiovascular, echocardiography, cardiology, infection, thrombus, covid-19

## Abstract

Severe acute respiratory syndrome coronavirus 2 (SARS-CoV-2) (COVID-19) is a viral disease that predominantly affects the respiratory system, but extrapulmonary manifestations have been increasingly reported over the course of the pandemic. Common extrapulmonary manifestations include the gastrointestinal, cardiovascular, and neurological systems, such as diarrhea, rashes, loss of smell/taste, myalgia, acute kidney injury, cardiac arrhythmias, or heart failure. COVID-19 infection is associated with an increased risk of thromboembolic events, especially in the setting of severe disease. We present a case of a 42-year-old female who recently tested positive for COVID-19 infection and presented to the clinic with complaints of palpitations that started after her diagnosis. An electrocardiogram done in the clinic showed sinus rhythm, and the patient was placed on an event monitor, which showed no evidence of tachyarrhythmia. A transthoracic echocardiogram (TTE) done as part of the workup showed a large thrombus in the right ventricular outflow tract attached to the ventricular side of the pulmonic valve. The patient was started on a therapeutic dose of apixaban at 10 mg twice a day (BID) for seven days and 5 mg twice a day afterward.

## Introduction

Initially recognized as a virus related to respiratory disease, severe acute respiratory syndrome coronavirus 2 (SARS-CoV-2) has found numerous tissue tropisms, thus acquiring an ability to cause systemic damage [1,2]. Rare complications involving the cardiovascular system were found in 9.3% of confirmed cases, with atrial fibrillation and heart failure being the most common [3]. Surpassing the first month of infection, individuals with an active COVID-19 infection have an increased incidence of cardiovascular complications, including thromboembolic disease [4]. There has been growing evidence of COVID-19 creating a prothrombotic environment leading to venous or arterial thromboembolism [5]. It is postulated that vascular thromboembolism arises from severe inflammation, hypoxia, and diffuse intravascular coagulation [5-8]. We present a case of a patient who initially presented with palpitations after recently testing positive for COVID-19. A right ventricular outflow tract thrombus was discovered on a transthoracic echocardiogram (TTE). The workup for other causes of hypercoagulation done prior to starting therapeutic anticoagulation was negative, and her prior COVID-19 infection was thought to be the most probable cause of thrombus formation. This is one of few cases that reports an intracardiac thrombus arising from a mild COVID-19 infection.

## Case presentation

A 42-year-old female with a past medical history significant for deep vein thrombosis (DVT) during pregnancy, gastroesophageal reflux disease, rheumatoid arthritis, and depression presented to an outpatient clinic with a chief complaint of palpitations in the past 24 hours. She reported feelings of general malaise and minor body aches but was afebrile and denied chills, chest pain, dyspnea, and orthopnea. The palpitations were not associated with syncope or presyncope. On physical examination, the patient’s heart rate and rhythm were normal with no murmurs, rubs, or gallops. Pulmonary effort and breath sounds were normal with no stridor, wheezing, or rales. She tested positive for COVID-19 six days prior through a home testing kit and still tested positive once she arrived at the hospital. The patient previously received two total Pfizer COVID-19 vaccinations, with the most recent one being one year and eight months prior. The patient reports that she had never received a booster vaccination. A complete blood count (CBC) with differential was completed on the day of the patient’s initial visit. The CBC showed elevated neutrophils and monocytes with decreased lymphocytes (Table 1). Her basic laboratory results including thyroid-stimulating hormone were within normal limits. A chest X-ray was performed, and the findings were unremarkable. At this time, an event monitor was placed by the physician, laboratory examinations were sent, and after discussing indications for urgent follow-up, she was discharged.

**Table 1:** Results of complete blood count with differential taken on the day of the initial visit *, abnormal value; Abs, absolute; Hgb, hemoglobin concentration; uL, microliters; g/dL, grams/deciliter; fL, femtoliters; pg, picograms

Component	Results	Normal range	Units
White blood cell	8.70	4.00-10.00	10^3^/uL
Red blood cell	4.45	4.10-5.30	10^6^/uL
Hemoglobin	12.1	11.5-15.5	g/dL
Hematocrit	36.4	36.0-45.0	%
Mean cell volume	81.9	80.0-96.0	fL
Mean cell hemoglobin	27.3	27.0-33.0	pg
Mean cell (Hgb)	33.3	32.0-36.0	g/dL
Red cell distribution width	13.7	11.5-14.5	%
Platelet count	267.0	150.0-400.0	10^3^/uL
*Abs neutrophil	7.44	1.80-7.00	10^3^/uL
*Abs lymphocyte	0.31	1.20-4.00	10^3^/uL
*Abs monocyte	0.93	0.00-0.80	10^3^/uL
Abs eosinophil	0.02	0.00-0.50	10^3^/uL
Abs basophil	0.01	0.00-0.20	10^3^/uL
Neutrophil	85.0	40.0-60.0	%
Lymphocyte	4.0	20.0-40.0	%
Monocyte	11.0	2.0-8.0	%
Eosinophil	0.0	1.0-4.0	%
Basophil	0.0	0.5-1.0	%

**Figure 1:**
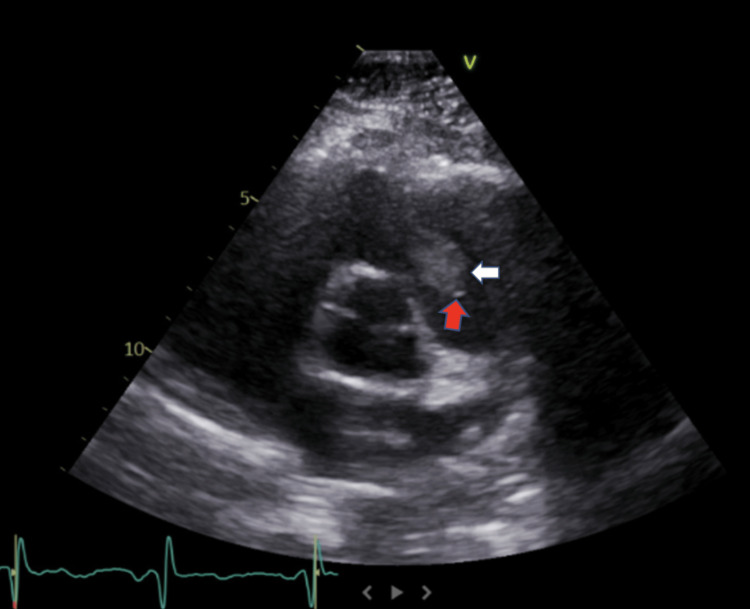
Parasternal long-axis view echocardiography of the right ventricle The white arrow shows the thrombus found in the right ventricle attached to the pulmonic valve (red arrow).

**Figure 2:**
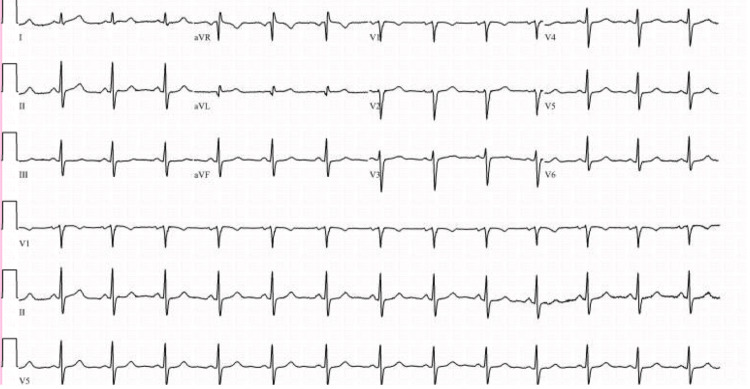
Electrocardiogram showing normal sinus rhythm

One week later, the patient returned to the clinic for a TTE as part of her workup for palpitations. She denied any fever, dyspnea, or cough, but she had a malaise since she was last seen. The TTE revealed a large thrombus in the right ventricular outflow tract attached to the ventricular side of the pulmonic valve (Figure 1). Pulmonic valve regurgitation was also present. The left and right ventricles sizes, systolic function, and left ventricular ejection fraction were all normal (69%) (normal range: 55%-70%). An electrocardiogram was performed, which showed sinus rhythm (Figure 2). The hypercoagulability panel done at the follow-up presentation was all within normal limits, except for a slightly elevated D-dimer (Table 2). She started a therapeutic dose of apixaban at 10 mg twice a day (BID) for seven days and 5 mg BID afterward.

**Table 2:** Hypercoagulability panel taken one week after the initial presentation with palpitations *, abnormal value; APTT-LA, activated partial thromboplastin time-lupus anticoagulant; ng/mL, nanograms/milliliter; μmol/L, micrograms/liter; IU/dL, international units/deciliter

Laboratory tests	Results	Normal range	Units
Antithrombin III activity	93	80-120	%
APTT-LA (lupus-sensitive reagent)	27.3	20-39	Seconds
*D-dimer	588	220-500	ng/mL
Factor VIII activity	108	50-150	%
Homocysteine	9.2	5-12	μmol/L
Protein C activity	97	68-162	IU/dL
Protein S activity	56	49-130	IU/dL
Russell viper venom time (dilute)	38	29-42	Seconds

At her one-week follow-up visit, the patient underwent a repeat TTE, which showed a stable thrombus that was similar in size and was still adherent to the pulmonic valve. Her symptoms improved, as she reported having only one episode of palpitations since her initial visit and the event monitor showed no tachyarrhythmia. The patient reports having been compliant with the anticoagulation medication, and she was advised to continue apixaban 5 mg BID. The patient reported that she had started exercising regularly and modified her diet to decrease refined carbohydrate and salt consumption, both of which she was advised to continue.

## Discussion

Falling under the beta coronavirus family, SARS-CoV-2 follows the angiotensin-converting enzyme 2 (ACE2) receptor mechanism, like its related counterpart, SARS-CoV-1 [1]. Genomic analysis of the novel virus sheds light on the pathophysiology of SARS-CoV-2 affecting other organs while excluding pulmonary symptoms [1]. The lungs are thought to be the main target, but ACE2 receptor expression in the kidneys, heart, and bladder explains multi-organ involvement from infection [1]. Studies have also found expression of nicotinamide adenine dinucleotide phosphate (NADPH) oxidase isozymes 2, 4, and 5 (NOX2, NOX4, and NOX5) in human cardiac microvascular endothelium [9]. These enzymes play a role in producing reactive oxygen species (ROS), and over-activation may contribute to microvascular endothelial dysfunction [9]. Myocardial infarction, heart failure, fibrosis, and microvascular permeability have correlated with increased endothelial expression of NOX2 and NOX5 [9]. Oxidative stress leading to high ROS levels is key in prothrombotic and procoagulant activation [9]. Heart tissue taken from the left and right ventricles of deceased COVID-19 patients found intravascular thrombosis in the presence of prothrombotic proteins (tissue factor, factor VII, and factor XII) [9].

COVID-19 infection increases the likelihood that patients will experience thrombotic and thromboembolic events [7]. These events occur because of the hypercoagulable environment that COVID-19 infection stimulates [7]. Prolonged activated partial thromboplastin time (PT/APTT) and increased fibrin(ogen) degradation products such as D-dimer are two coagulation abnormalities that are commonly seen in COVID-19 [7]. Antithrombin and protein S activity are usually found to be decreased in COVID-19 [7]. Interestingly, D-dimer was the only abnormal value found in the patient. We believe that the delay in collecting the patient’s blood for analysis was the reason for the normal findings of hypercoagulability markers. Transient changes in protein activity with a prolonged elevation in D-dimer are consistent with the pathophysiological changes associated with COVID-19. One differential that was considered was homocystinuria. However, procoagulant factors such as protein C and protein S, and homocysteine were within normal limits. Excessive homocysteine increases the probability of thrombus and ischemia because of prothrombotic environments. Thus, we were able to rule out metabolic dysfunction as the cause of the thrombus.

To activate the coagulation cascade, immune cells are an integral part of thrombosis. Data has been reported on the function of non-myeloid inflammatory cells such as T lymphocytes and monocytes in initiating a thrombotic response [10]. T lymphocytes have been found to regulate the prothrombotic action of activated neutrophils in the process of fibrin formation [10]. A significant association of thromboembolism was found in patients with an increased neutrophil-to-lymphocyte ratio [10]. However, using individual immune cell counts did not show a correlation with thrombosis [10]. Neutrophils promote thrombus growth by presenting proteases and coagulation factors and serving as a scaffold for fibrin polymerization [10]. The role of lymphocytes in thrombogenesis is unclear, but it is postulated that they modulate innate immune cell activity during thrombus resolution [10]. Our patient’s CBC supports the hypothesis that an increased neutrophil-to-lymphocyte ratio promotes thrombus generation.

Our patient can be categorized as mildly symptomatic, which is defined as an individual who has tested positive for COVID-19 and shows symptoms of malaise, fever, and cough but does not have shortness of breath, dyspnea, or abnormal chest imaging [11]. In contrast, patients who are critically ill are more susceptible to systemic dysfunction, including multiple organ failure, respiratory failure, or septic shock [11]. Over the course of the pandemic, reports of cardiovascular involvement in a COVID-19 infection had increased. However, these were associated with a more severe infection than the one seen in our patient. In addition, there is increasing evidence that associates COVID-19 infection with an increased risk of thromboembolic events, but these are mainly associated with more severe symptomatic cases or with patients who have significant preexisting risk of developing thromboembolism. Studies show an increased incidence of thrombotic complications in severe COVID-19 infections with an incidence of 31% (95% confidence interval: 20%-41%) in critically ill intensive care unit patients [6]. This leads us to believe that our case highlights a rare finding of a patient with a mild COVID-19 infection who developed a right heart thrombus (RHT).

RHT is a rare form of venous thromboembolism (VTE) that is a potentially life-threatening condition and is underreported in association with COVID-19. Dilated cardiomyopathy is associated with right and left ventricle dilation and reduced systolic function [12]. Biventricular stasis promotes thrombus formation commonly in the left ventricle, followed by the right ventricle [12]. Incidence of a ventricular thrombus is uncommon at 4% in patients with pulmonary embolism, but mortality rates reached as high as 29% [13]. Cardiovascular risk factors or a history of venous thromboembolism (VTE) increases the risk of future VTE [8]. Recent studies correlated an increased risk of VTE with concomitant pulmonary embolism or deep vein thrombosis (DVT). Our patient had a 10-year history of DVT originating in the profunda femoris vein, which had been treated with enoxaparin sodium. Differentials of pulmonary embolism and embolic DVT were ruled out through a TTE with contrast. In 2013, our patient had a DVT provoked by pregnancy. Workup at the time of pregnancy revealed a factor V Leiden mutation. A mutated factor V Leiden is resistant to the anticoagulant protein C, which increases the risk for thrombosis. With a history of DVT, a family history of DVT, and a genetic mutation, it was believed that COVID-19 exacerbated the patient’s hypercoagulability. DVT is commonly associated with type A thrombi [14]. These are characterized as being highly mobile and morphologically serpiginous [14]. Often in transit, type A thrombus was not the case in our patient, as TTE showed a stable right heart thrombus attached to the pulmonic valve [14]. TTE is the standard practice in screening for cardiac thrombi and is the most used imaging test in the United States [15,16]. When using TTE for clinically indicated patients with ventricular thrombus, sensitivity and positive predictive value increased to 60% and 75%, respectively, and the use of contrast reduced rates of false positives and false negatives [15]. Although TTE is widely used to determine cardiac function and structure, a thrombus is rarely the primary indication, possibly leading to misinterpretation [17]. Thus, the combination of low contrast usage in TTE with RHT as the primary indication is relatively rare and puts patients at risk for life-threatening complications.

One of the most common treatments for thrombosis is the administration of anticoagulant medication. However, in more severe cases, surgical procedures may be indicated. Larger thrombi or increased risk for mobilization may indicate surgical intervention, whereas smaller, immobile thrombi can be safely controlled with anticoagulation [18]. Traditionally, warfarin was the preferred anticoagulation agent, but studies have shown direct oral anticoagulants are non-inferior to warfarin in the treatment of intracardiac thrombus [19]. In our case, the decision was made to administer apixaban, a direct oral anticoagulant that directly inhibits factor Xa, which cleaves prothrombin to generate thrombin in the penultimate step in the coagulation cascade leading to fibrin and clot formation [20]. Normal propagation of the cascade is associated with large amounts of thrombin being formed at the site of injury, but apixaban inhibits this process [20]. Finally, fibrin formation normally occurs when activated thrombin cleaves fibrinogen, but since apixaban is inhibiting thrombin in the prior step, the cleavage of fibrinogen is disrupted. Upon the discovery of the thrombus, our patient was started on a therapeutic dose of apixaban 10 mg BID for seven days and tapered to 5 mg BID after. A follow-up TTE after one week showed some reduction in the size of the thrombus but marked improvement in her symptoms. The patient was scheduled for a regular follow-up, and a TTE was planned after six to eight weeks of treatment.

## Conclusions

This case highlights a rare occurrence of a mild COVID-19 infection followed by the discovery of an intracardiac thrombus. A thrombus attached to the pulmonic valve was detected on TTE. We believe that even a mild infection may support a hypercoagulable state, increasing the risk of cardiovascular involvement. This case emphasizes the importance of early detection and management of cardiac thrombus as a possible complication of COVID-19 infection in a patient without severe symptomatic infection. Clinical trials are needed to further investigate the possible causes of intracardiac thrombus resulting from mild infections. There is a need for clear guidelines regarding follow-up imaging after the diagnosis and treatment of intracardiac thrombus.
